# In Silico Insights towards the Identification of SARS-CoV-2 NSP13 Helicase Druggable Pockets

**DOI:** 10.3390/biom12040482

**Published:** 2022-03-22

**Authors:** Federico Ricci, Rosaria Gitto, Giovanna Pitasi, Laura De Luca

**Affiliations:** Department of Chemical, Biological, Pharmaceutical, and Environmental Sciences, University of Messina, 98166 Messina, Italy; federico.ricci@unime.it (F.R.); rosaria.gitto@unime.it (R.G.); giovannapitasi@libero.it (G.P.)

**Keywords:** COVID-19, Nsp13, binding site prediction, protein structure, FTMap, SiteMap, Fpocket, LigandScout software packages

## Abstract

The merging of distinct computational approaches has become a powerful strategy for discovering new biologically active compounds. By using molecular modeling, significant efforts have recently resulted in the development of new molecules, demonstrating high efficiency in reducing the replication of severe acute respiratory coronavirus 2 (SARS-CoV-2), the agent responsible for the COVID-19 pandemic. We have focused our interest on non-structural protein Nsp13 (NTPase/helicase), as a crucial protein, embedded in the replication–transcription complex (RTC), that controls the virus life cycle. To assist in the identification of the most druggable surfaces of Nsps13, we applied a combination of four computational tools: FTMap, SiteMap, Fpocket and LigandScout. These software packages explored the binding sites for different three-dimensional structures of RTC complexes (PDB codes: 6XEZ, 7CXM, 7CXN), thus, detecting several hot spots, that were clustered to obtain ensemble consensus sites, through a combination of four different approaches. The comparison of data provided new insights about putative druggable sites that might be employed for further docking simulations on druggable surfaces of Nsps13, in a scenario of repurposing drugs.

## 1. Introduction

The severe acute respiratory coronavirus 2 (SARS-CoV-2) infection is responsible for the global COVID-19 disease, which emerged in late 2019, in Wuhan, Hubei, China [1]. COVID-19 mortality is due to acute respiratory syndrome and excessive release of cytokine and chemokines [2]. Extensive efforts have led to the development of vaccines and monoclonal antibody therapies; moreover, several potential therapeutics have entered clinical trials [3,4]. All these therapeutic interventions might offer effective opportunities to combat SARS-CoV-2 emerging variants that could evade the currently employed therapies. SARS-CoV-2 belongs to the group of coronaviruses (named CoVs) [5], which are enveloped and positive single-stranded RNA viruses, having, on their surface, the spike proteins (S-proteins) capable to interact with angiotensin-converting enzyme 2 (ACE-2) receptors, on the human host cell membrane. The knowledge of the SARS-CoV-2 life cycle was addressed to develop new potential agents, targeting virus entry and fusion and replication processes [6].

The SARS-CoV-2 genome encodes structural and accessory proteins, as well as non-structural proteins (Nsps), controlling RNA transcription, replication and immune evasion [7]. All sixteen Nsps are generated by the cleavage of polyproteins, that are expressed by the open reading frame ORF1ab. The viral cysteine proteases (3CLpro; Nsp5) and the papain-like protease (PLpro; Nsp3) are responsible for the above-mentioned proteolytic process. Moreover, the Nsps include Nsp12 (RNA-dependent RNA polymerase), Nsp13 (NTPase/helicase) and Nsp14 (endoribonuclease), as crucial proteins composing the replication–transcription complex (RTC), which controls the virus life cycle. Among them, Nsp13 possesses multiple enzymatic activities, essential for the replication of the viral genome, so that it represents a valuable drug target for the development of therapeutics towards SARS-CoV-2 [8,9]. Specifically, Nsp13 performs the 5′-capping of the viral RNA by triphosphatase activity, leading to the hydrolysis of the 5-triphosphate group in diphosphate-RNA, as the initial step that induces the 5′-cap modification of RNA (also named RNA capping process); the helicase activity of Nsp13 also performs the unwinding of the RNA helices in an ATP-dependent manner. As a structural protein, essential for virus replication and proliferation, the helicase forms complexes with other Nsps (Nsp7, Nsp8, Nsp9, Nsp12, and Nsp14), as well as host factors. Overall, Nsp13 facilitates the correct folding and replication of viral RNA.

The viral helicases are extensively explored for their applications, as valuable targets for the development of efficacious therapies [10,11], as already addressed for RNA-dependent RNA polymerases. It is noteworthy that the Nsp13 sequence is highly conserved among SARS-like coronaviruses (up to 99.8% sequence identity) [12], thus, revealing it as a promising target for pan-coronavirus therapeutics.

To evaluate the druggability of Nsp13 protein and accelerate the drug design of Nsp13 inhibitors as antiviral agents against SARS-CoV-2, we have studied plausible druggable pockets on the Nsp13 dimer, using computational methods in place of expensive and time-consuming experimental procedures, such as alanine scanning or NMR-based analyses. It is well known that there are different approaches to determine the druggability of novel and highly challenging pharmaceutical targets; actually, these methods might be based on experimental or theoretical procedures [13]. Particularly, the computational approaches are based on the analysis of various pocket properties, including hydrophobicity, size, compactness, hydrogen-bond donor and -acceptor surface areas, and amino acid composition in pockets. Thus, evaluating the druggability of proteins, with respect to conventional druglike compounds, is considered a mature technology that can perform such assessments with reliability.

In this work, to assess the druggability of binding pockets on currently available Nsp13 structures, we focused our interest on the in-silico identification of the most plausible hot spot residues that might represent the residues having significant contribution to the binding free energy on the Nsp13 dimer. In fact, the hot spot residues are generally expected to significantly impact in protein binding interaction [14,15]. Therefore, the identification of hot spots could offer amenable binding sites for efficacious drugs, capable of interfering in the virus life cycle, thus, highlighting the best druggable binging pockets.

To perform the planned computational analysis, we studied three Nsp13 structures available on RCSB PDB databases, and these proteins were selected as unbound structures, considering that the ligand-free proteins generally furnish valid druggability assessment for most targets, for which the presence of ligands could induce conformational changes in binding pockets.

For each selected Nsp13 structure, we performed an analysis of the protein structure druggability, considering four different approaches, using docking-based, grid-based, and geometry-based algorithms. Then, we combined these results, leading to the identification of different ensemble consensus sites for potential ligands suitable for future computational studies, as well as drug design campaigns, focused on novel target Nsp13 protein to develop antiviral agents against SARS-CoV-2.

## 2. Materials and Methods

### 2.1. Protein Preparation

To perform our structural analysis, we selected Cryo-EM structures containing the SARS-CoV-2 Replication/Transcription complex (RTC) in order to sample Nsp13 conformations interacting with the other viral non-structural proteins (Nsp7, Nsp8, Nsp12) and the RNA. Thus, three RTC structures were downloaded from the protein data bank RCSB PDB (PDB codes: 6XEZ, 7CXM, 7CXN showing a resolution of 3.5 Å, 2.9 Å and 3.84 Å respectively) [16,17].

Actually, a further Cryo-EM structure containing Nsp13 is available on PDB (7CYQ) [18]; it consists of an extended SARS-CoV-2 replication and transcription complex (RTC) assembled by Nsp7-Nsp82-Nsp12-Nsp13_2_-RNA and a single RNA-binding protein, Nsp9 [18]. To perform our study, we chose to discard this structure as it contained several missing residues of Nsp13 and their reconstruction could have altered the prediction analysis.

From the three examined complexes, the two monomers Nsp13-1 (chain E) and Nsp13-2 (chain F) were extracted and treated separately as a single chain. Actually, these two monomers in the larger structure show a different orientation from each other.

Nsp13-1 and Nsp13-2 were extracted from the PDB 6XEZ and used as reference structures to align the corresponding monomers extracted from the other two structures (PDB 7CXM and 7CXN) by using Pymol (https://pymol.org, accessed on 25 February 2022) [19]. This alignment achieved two main purposes: the first one was to help the visualization and the superimposition of the results, the second one was to place the magnesium (Mg) ion, which constitutes a cofactor of the Nsp13 ATPase domain, for the two structures for which it is lacking (7CXM and 7CXN).

Successively, all the protein structures were prepared as follows: (i) the ligands were removed; (ii) missing side chains were predicted using Prime [20]; (iii) hydrogen atoms were added using the Maestro Protein preparation wizard [20].

The quality of the protein structures was determined by analyzing their Ramachandran Plots as implemented in VEGA program [21] (see Table S1 in Supplementary Material). Furthermore, in order to have information about the flexibility of the selected proteins, we calculated the RMSD values for the prepared protein structures considering all their atoms (7CXM and 7CXN) with respect to the reference protein structure 6XEZ. Therefore, we obtained 1.93 Å and 3.07 Å RMSD values for Nsp13-1 and 3.97 Å and 1.58 Å RMSD values for Nsp13-2 of 7CXM and 7CXN, respectively.

### 2.2. Binding Site Detection

Binding site prediction was carried out for the three Nsp13-1 and the three Nsp13-2 and for each protein conformation by employing the four programs (SiteMap, fpocket, FTMap, and LigandScout), which use structure-based algorithms for their calculations. To run the calculations, we used default parameters. A detailed overview of the four programs as well as a comparison of their performance are provided below.

**SiteMap** module, implemented in Schrödinger Suite 2020-3 [22], identifies putative binding sites in a protein using an energy-based algorithm, which performs three main sequential steps for defining plausible binding pockets as follows: (i) detection of the pockets; (ii) characterization of the pockets, and (iii) scoring of the cavities. The pocket detection step could be further subdivided into three distinct stages: in the first one, a 1 Å grid of points is built around the protein, and the grid points that overlap the protein atoms are discarded as well as the ones presenting a low relative enclosure score (<0.5 by default), which is the fraction of 110 radial rays traced from a grid point that hit the protein surface within a given distance (8 Å). Furthermore, a cutoff of −1.1 kcal/mol on the vdW interaction energy is applied to the grid points to reject enclosure points that establish contacts with unfavorable protein [22]. Then, a clustering of the site points into site groups is performed merging at least 15 site points (according to default settings) that are closer to each other until all the site points are examined; we discarded the point groups that do not respect these criteria. Subsequently, a further merge of site point groups is done to examine whether the closest points of two or more group are distanced in solvent exposed region less than 4 Å by default. Site groups with less than 100 site points are considered during this last merge. In the second step of the calculation, the just-detected sites were characterized using water probes, thus creating vdW and electric field grids to map hydrophilic regions, which were further subdivided into H-bond acceptor, H-bond donor and metal-binding regions; we also mapped hydrophobic regions and “neither/nor” regions, which represent regions having a mixed character or being far to the receptor surface [23]. Finally, the resulting sites are scored and ranked according to the Sitescore and by default the 5 top-ranked sites are returned to user. SiteMap also generates scores (SiteScore, Dscore) that were used to assess the druggability.

The following SiteMap’s Sitescore and Dscore scoring functions are found: (i) SiteScore = 0.0733 n1/2 + 0.6688 e-0.20 p; (ii) Dscore = 0.094 n1⁄2 + 0.60 e-0.324 p. The two scoring functions are the sum of the same contributions: (i) “n” represents the number of the site points of the site scored; (ii) “e” is the enclosure score; (iii) “p” is the hydrophilic score. The two scoring functions differ exclusively for the coefficient of the given contributions; in the first function both “n” and “p” are capped at 100, in the second function only “n” is capped at 100; this choice penalizes the sites rich of hydrophilic regions and prone to host charged ligands that might not be druglike. Based on these considerations, we chose SiteMap Dscore to rank the consensus sites that satisfied our selection criteria.

**Fpocket** (version 2.0) [24] is on open source program, whose geometry-based algorithm detects potential binding sites using Voronoi tessellation and sequential clustering steps. The Fpocket workflow can be described as follows: the protein heavy atom sets to the Voronoi, a program of Qhull package, which returns the list of the coordinates of the Voronoi vertices to Fpocket, as well as of the atomic neighbors and vertex neighbors. For each Voronoi vertex a so-called alpha sphere contacting a least four atoms is traced. Then, the alpha spheres are filtered retaining by default only the ones having a radius between 3 and 6 Å; they can be labeled as apolar or polar spheres. Subsequently, alpha spheres are submitted to a first distance clustering using the vertex neighbors list as input (default distance 1.74 Å); then, for each cluster the center of mass is calculated and proximal centers of mass are further merged. Finally, a multiple linkage clustering is performed and clusters are merged if a certain number of alpha spheres are near to a certain number of alpha spheres of another.

Sites are then scored and ranked according to the Fpocket SiteScore. In the Fpocket, Site score take into account the following parameters: (i) the normalized number of alpha spheres; (ii) the normalized mean local hydrophobic density; (iii) the normalized proportion of apolar alpha sphere; (iv) the polarity score (sum of polarity over all amino acids involved in a given pocket using a binary scheme, e.g., 1 for polar, 0 for non-polar); (v) the alpha sphere density, which is defined as the mean value of all alpha sphere pair to pair distances in the binding pocket. Druggability score considers the normalized mean local hydrophobic density and polarity score too, but a new term (hydrophobicity score) is introduced and the coefficients of the three parameters are based on a validation study conducted by Schimdtke et al. [25].

Similarly to SiteMap, Fpocket Site score is unable to distinguish pockets capable of hosting druglike molecules to the ones able to host only polar ligands [25]. For this reason, Fpocket Dscore considered during consensus site ranking, giving a higher-ranking position to pockets in which a correspondence between the druggability score values reported by the two software was present.

According to a benchmark study conducted in 9900 apo proteins and 5416 protein ligand complexes, SiteMap and Fpocket provided comparable results, especially in terms of site ranking; however, SiteMap attempted to predict slightly bigger pockets [23]. Recently, a set of druggable and non-druggable cavities was employed to evaluate both software, this study revealed that Fpocket is one order of magnitude more efficient than SiteMap [26]. Nonetheless, we believe that the combination of software, which present two very distinct methods (energy-based and geometry-based respectively) as well as a different scoring function, could be useful to estimate putative binding pockets in unknown targets for which few data are available in literature.

**FTMap** web server (https://ftmap.bu.edu, accessed on 11 January 2022) is an open-source mapping web-server for binding hot spots identification that employs 16 small molecules (probes) differing in size, shape and polarity to define hot spots through conformational and spatial searches, clustering procedures and evaluation of the interaction energy of the probes on a dense grid using an empirical energy function that includes a continuum electrostatic term.

Differently from SiteMap and Fpocket, FTmap recognizes hot spots, which are regions of binding site/receptor in which high binding affinity for small molecule probes are displayed during X-Ray or NMR experiments. Complementary to our study, we employed the results obtained from FTMap to highlight hot spot, which can be encountered in the cavities previously detected via SiteMap and Fpocket. FTMap accounts druggability according to the number of probe clusters that are present in a consensus cluster (i.e., FTMap hotspots). These data are collected in Table S2, even if they were not considered for pocket selection or ranking because they are not relatable to the previous druggability scoring functions described for the other software.

**Ligandscout** (version 4.4.8) [27] presents a “Calculate pockets” function, which detects accessible cavities placing a grid surface around the protein and creating pockets on the basis of two adjustable parameters: the buriedness and the threshold. The buriedness value is calculated for each grid point and several clusters of the grid points are performed to define the cavities. Finally, isosurfaces are employed for rendering pockets. At the end of the process, Ligandscout accounts the number of grid pockets of each cluster/pocket to determine whether a pocket is druggable or not. The buriedness parameter defines the minimum value of buriedness (default value 0.50) required for considering, or not, a grid point during the clustering steps. The threshold parameter (default value 0.30) specifies instead the minimum cluster size to consider whether a pocket is druggable or not.

Overall, by using Maestro GUI, which is the graphical user interface (GUI) for all of Schrödinger’s products, we collected the results of the calculations performed by each program, thus generating overlapping sites that are reported in Table S2 in Supplementary Material. Table S2 also displays all data concerning the druggability that were collected by each software; the following criteria were applied for selecting and ranking the plausible ensemble-consensus sites:

− we considered the pockets that overlapped in the same region for the four employed programs reasonable, as well as found on two of three selected PDBs.
− we discarded the pockets that were considered not plausible by one of the four software on two of three selected PDBs.

## 3. Results and Discussion

It is well known that the assessment of druggability of specific regions of protein surfaces may offer great opportunity to identify small molecule inhibitors, capable of developing promising therapeutic candidates. Distinct enzymes involved in the SARS-CoV-2 replication are characterized by shallow surfaces, thus, resulting in challenges for the discovery process, which employs computational approaches, aimed at searching druggable binding sites. To detect the presence of druggable areas suitable for the binding of inhibitors, it is crucial to exploit the binding hot spots at the viral protein surfaces. Here, we employed distinct computational approaches to leverage protein surfaces that are major contributors for the binding of ligands, capable of inhibiting the activity of helicase Nsp13 of SARS-CoV-2, thus, reducing the virus replication and infectious capacity.

To gain insights into the protein hot spots involved in the binding process, all the potential druggable sites of the Nsp13 protein were explored. The identification of binding sites and the assessment of their druggability was conducted by computational methods, based on knowledge of the Nsp13 structure. In this work, we applied three different conformations of the two chains of the Nsp13 dimer (the two subunits named Nsp13-1 and Nsp13-2), assembled in the replication/transcription complex (RTC) of SARS-CoV-2.

For our computational studies, we selected the dimer Nsp13 that is present in three different RTC complexes (PDB codes: 6XEZ, 7CXM, 7CXN) [8,17,18].

Specifically, we have investigated the Nsp13 dimer by using four different software packages: FTMap, SiteMap, Fpocket and LigandScout [27]; thus, merging four different methods for the analysis of the protein structure druggability (docking-based, grid-based, and geometry-based algorithms). For each prediction we detected hot spots that were visualized on Maestro GUI. The overlapping hot spots were merged by means of visual inspection; then, the putative binding sites were defined and ranked, using the criteria that were described in more detail in the Section 2.

For each approach, the different structural analyses for sampling binding sites are discussed in the following sections.

### 3.1. SiteMap Analysis

SiteMap is an application implemented in the Maestro suite, whose algorithm is inspired on the Goodford’s GRID one. By employing the default settings (see Materials and Methods), in step 1 of our study, we identified the main hot spots for each Cryo-EM structure (PDB codes: 6XEZ, 7CXM, 7CXN) [8,17,18] and measured their Druggability score (Dscore). Binding pockets with Dscores lower than 0.83 are predicted “undruggable”, pockets with Dscores above 0.98 are predicted “druggable”, and Dscores between these thresholds indicate sites that are difficult to drug [22]. We considered druggable sites only as the pockets that showed, in at least two of the three monomers, a Dscore > 0.9 (see Table S2 in Supplementary Material).

**Figure 1:**
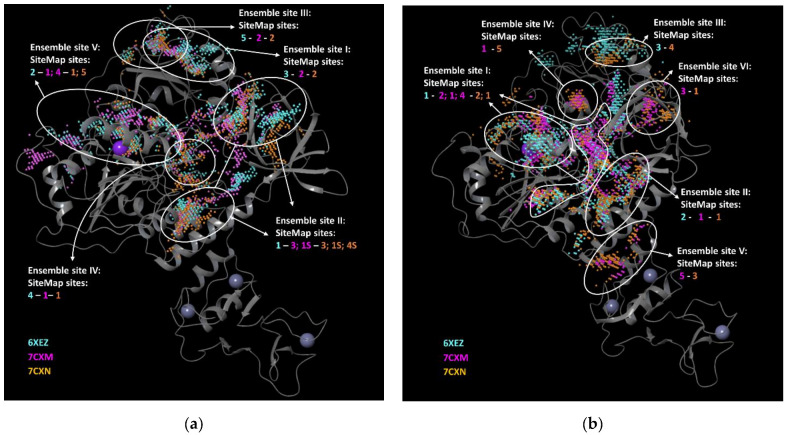
Ensemble binding pockets detected by SiteMap for an aligned ensemble of three (**a**) Nsp13-1 and (**b**) Nsp13-2. SiteMap detected ensemble-binding pockets that overlaid on an exemplar structure (6XEZ). Spheres corresponding to cavities detected by SiteMap are shown in cyan for Nsp13 of 6XEZ, in magenta for Nsp13 of 7CXM and in orange for 7CXN. Shared regions defining the ensemble sites are highlighted by the white outlines which also report the overlapping site in the text boxes. These images were prepared by Maestro GUI [20].

According to the pocket analysis focused on the Nsp13-1, we identified five so-called ensemble sites (Ensemble I–V), based on the clustering of the common parts of the SiteMap results, for the three employed structures (6XEZ, 7CXM, 7CXN) (Figure 1a and Table S2). For the three Nsp13-2 monomers, from 6XEZ, 7CXM, 7CXN structures, SiteMap predicted hot spots that were clustered in six ensemble sites (Ensemble I–VI) (Figure 1b, Table S2).

### 3.2. Fpocket Analysis

Then, we performed the pocket analysis of the Nsp13 dimer by using Fpocket tool, which is an open-source program, capable of generating potential ligand-binding pockets. Specifically, Fpocket possesses a geometry-based algorithm that makes use of Voronoi tessellation and alpha spheres to detect pockets and report different properties (such as pocket score, druggability score, volume, etc.) of determined pockets. For the Nsp13-1 monomer, this program predicted three distinct sets of binding pockets (i.e., forty-eight for 6XEZ, fifty-one for 7CXM, and forty-eight for 7CXN). The Fpocket analysis also identified forty-six, forty-five, and fifty-five pockets for the three above-mentioned structures 6XEZ, 7CXM, 7CXN on the Nsp13-2 monomer.

**Figure 2:**
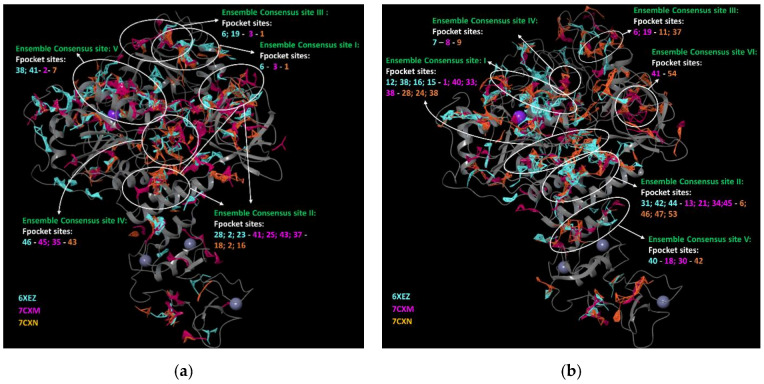
Ensemble consensus binding pockets by Fpocket for an aligned ensemble of three (**a**) Nsp13-1 and (**b**) Nsp13-2; overlaid on an exemplar structure (PDB 6XEZ). The images were prepared by Maestro GUI [20].

Interestingly, Fpocket was able to find sites that possessed overlapping regions, with all the sites detected by SiteMap, thus, clustering five Ensemble consensus sites I–V for Nsp13-1 and six Ensemble consensus sites I–VI for Nsp13-2 (Figure 2).

These sites were found by Fpocket for all the PDBs studied, with the exception of Ensemble consensus sites III and VI of Nsp13-2, which were found for two out of the three employed PDB structures.

Furthermore, F-pockets provided a druggability score between 0 and 1; a low score implies that druglike molecules are not likely to bind to the pocket, while values higher than 0.5 (the threshold value) indicated that a binding interaction could be possible.

With the exception of site II of Nsp13-1 (best druggability score of 0.446 in PDB 7CXN), and site IV of Nsp13-2 (best druggability score 0.047 in PDB 7CXN), the Ensemble Consensus sites identified the most plausible site for one of the three PDBs used. Fpocket was able to report sites having a Dscore ≥ 0.5, thus, providing us results partially in accordance with those given by SiteMap. A comparison of Fpocket results with those reported by the other programs is visible in Table S2.

### 3.3. FTMap Analysis

The third program that we used was FTMap. This method distributes small organic probe molecules, of varying size, shape, and polarity, on a macromolecule surface, thus, finding the most favorable positions for each probe type; then it clusters the probes and ranks the clusters on the basis of their average energy.

FTMap uses 16 organic molecules as probes (ethanol, isopropanol, isobutanol, acetone, acetaldehyde, dimethyl ether, cyclohexane, ethane, acetonitrile, urea, methylamine, phenol, benzaldehyde, benzene, acetamide, and N,Ndimethylformamide). Regions that bind several different probe clusters are called consensus sites, and the site containing the largest number of probe clusters is considered the main hot spot [28].

Unlike SiteMap and Fpocket, FTMap has no druggability score, so we compared the ensemble consensus sites found previously with the clusters obtained by FTMap.

For Nsp13-1 and Nsp13-2, FTMap detected different cross-clusters (e.g., binding sites), indicated as follows: (i) 14 for the PDB 6XEZ, 13 for the PDB 7CXM and 14 for the PDB 7CXN for Nsp13-1; (ii) 15 for the PDB 6XEZ, 10 for the PDB 7CXM and 16 for the PDB 7CXN for Nsp13-2.

**Figure 3:**
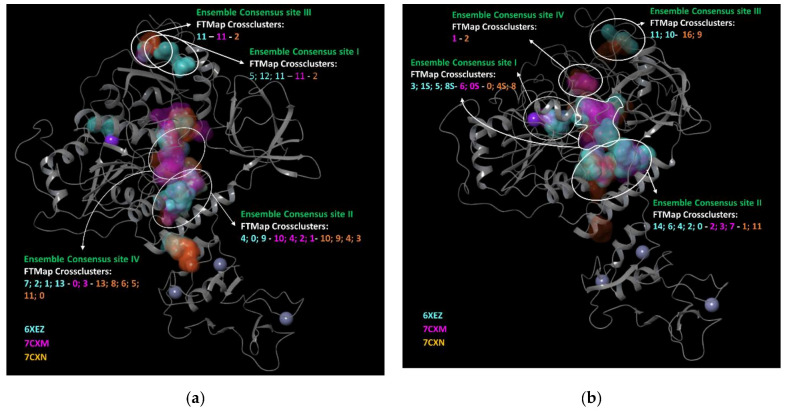
Ensemble consensus binding pockets predicted by FTMap for an aligned ensemble of three (**a**) Nsp13-1 and (**b**) Nsp13-2; overlaid on an exemplar structure (pdb code 6XEZ). Clustered probes are shown in cyan for nsp13 of 6XEZ, in magenta for nsp13 of 7CXM and in orange for 7CXN. The images were prepared by Maestro GUI [20].

FTMap was able to identify small binding pockets, which were fused to generate wider binding pockets; then, we selected only the pockets that overlapped the same region, on two of the three employed PDBs, and that overlapped the sites encountered with SiteMap and Fpocket (see in Table S2). Thus, we discarded the Ensemble Consensus pocket site V and reduced the area of the Ensemble Consensus site II for Nsp13-1. The combination of the Ensemble Consensus sites for Nsp13-2 resulted in four final pocket sites (I–IV) as showed in Figure 3.

### 3.4. LigandScout Analysis

**Figure 4:**
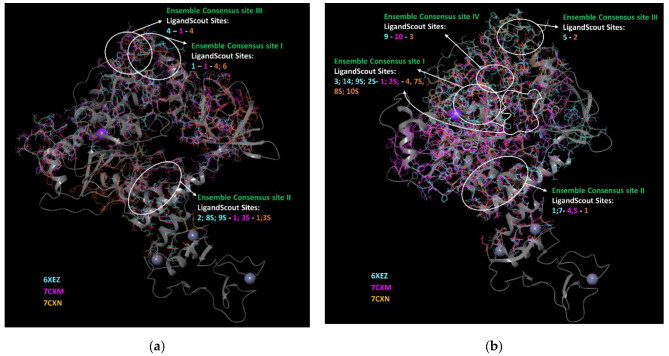
Ensemble consensus binding pockets predicted by LigandScout for an aligned ensemble of three (**a**) Nsp13-1 and (**b**) Nsp13-2; overlaid on an exemplar structure (PDB code 6XEZ). The images were prepared by Maestro GUI [20].

Finally, we included the LigandScout “Calculate pockets” algorithm in our analysis. By means of LigandScout, we identified a number of eligible druggable pockets. We saved the environment of the pocket as identified by the program and superimposed them to previous results, achieved by other employed programs (*cfr*. supra); finally, we analyzed whether they surrounded some of our ensemble-consensus sites. This step reduced them to three more plausible Ensemble Consensus sites (I–III) for Nsp13-1, whereas it keeps four Ensemble Consensus sites (I–IV) for Nsp13-2 (Figure 4). Ligandscout does not assess druggability through any scoring function, but analyzes geometrical properties; however, it reports if one identified pocket should be druggable or not. These data, related to our ensemble-consensus pocket, are reported in Table S2 but were not considered during the analysis.

### 3.5. Data Collection

**Figure 5:**
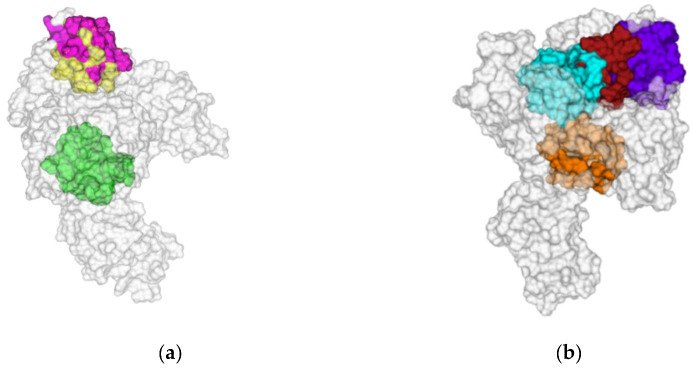
Ensemble consensus sites, highlighted as different color surfaces, for (**a**) Nsp13-1 and (**b**) Nsp13-2. The images were prepared by Pymol [19].

Collecting the results obtained by the above-reported sequential application of four distinct procedures, we have detected several putative binding sites that might be druggable; in detail, we detected three and four putative binding sites for Nsp13-1 and Nsp13-2, highlighted in Figure 5 as different color surfaces. For Nsp13-1, we represented (i) in yellow, the Ensemble Consensus site I, (ii) in green, the Ensemble Consensus site II and (iii) in magenta, the Ensemble Consensus site II. For Nsp13-2, we displayed (i) in cyan, the Ensemble Consensus site I, (ii) in orange, the Ensemble Consensus site II, (iii) in violet, the Ensemble Consensus site III and (iv) in purple, the Ensemble Consensus site IV. A more comprehensive description of each binding site is reported in Table S3 (see Supplementary Material), in which we described all residues forming the detected druggable binding sites.

It is interesting to note that some of the obtained binding sites are in good agreement with findings previously reported in the literature. In detail, the ensemble consensus site I of Nsp13-2 was found to be in accordance with the nucleotide/ATP binding site, described in the work of Newman et al. [29]. In this work, X-ray crystallographic fragment screening of Nsp13 showed that 15 fragments were bound in positions overlapping with the ATP ribose and adenine moiety. Fragments gave interactions with amino acids His290, Lys320, Lys324, Arg443, and Glu540, the same residues that are reported in Ensemble Consensus site I of Nsp13-2 [29]. This site was also explored by a molecular docking analysis on the ATP binding site, performed by Andrew White et al. [8], which led to the identification of potential inhibitory compounds, many of them approved human drugs. These compounds gave interactions with amino acids present in Ensemble Consensus site I of Nsp13-2, such as Gly285, Gly287, Ser289, Ala316, Lys320, Glu375, Arg443, Gln537, Gly538, and Glu540 [8]. Furthermore, Sajjad Ahmad et al. reported that the amino acid residues Lys288, Ser289, Asp374, Glu375, Gln404 and Arg567, are responsible for the hydrolytic activity of NTP; therefore, they are part of the ATP binding site. It is noticeable that these residues are located at Ensemble Consensus site I of Nsp13-2. In addition, this work displays two other binding sites, defined as binding site C and binding site D, in which the crucial amino acid residues are Lys139, Glu143, Phe145, Lys146, Asn179, Tyr180, Cys309, Asn361, Met378, Thr380, Val407, and Thr410. These sites are comparable to Ensemble Consensus site II of Nsp13-2, as found in previously reported studies [30].

Based on these above-reported data, we believe that our computational approach is characterized by satisfactory reliability. Moreover, our investigations resulted in the identification of other binding sites that are currently completely unexplored, thus, highlighting the novelty of our work.

## 4. Conclusions

For a better understanding of Nsp13/helicase of SARS-CoV-2, as a valuable target to develop efficient inhibitors, to combat the COVID-19 pandemic, we carried out a theoretical study to identify several hot spots on the protein surface. To perform this study, we used a sequential combination of four different tools, thus, describing ensemble consensus sites. The best outcome of this procedure was the detection of unexplored druggable sites, which might be leveraged to further in silico and in vitro drug discovery campaigns, aimed at identifying more efficient antiviral agents. In detail, our procedure allowed us to identify four sites, not yet reported in other studies: site I, II and III of Nsp13-1 and site III and IV of Nsp13-2. On the other hand, the identification of pockets, in agreement with sites reported in other studies and detected through other techniques (sites I and II of Nsp13-2), supplies a mutual confirmation of the best findings reported to date. We believe that a future perspective of this work might consist of performing docking and MD simulations of active compounds against Nsp13 helicase. This study would provide interesting insights about cavities binding active ligands, not yet co-crystalized.

## Data Availability

Not applicable.

## Supplementary Materials

The following supporting information can be downloaded at: https://www.mdpi.com/article/10.3390/biom12040482/s1, Table S1: Ramachandran plot for nsp13-1 and nsp13-2 extracted from PDB 6XEZ, 7CXM and 7CXN; Table S2: Ensemble-consensus binding site prediction report; Table S3: List of the protein residues forming the identified binding sites defined to include residues within 10 Å of any centroid of Fpocket results for each protein.
