# Supplementary material for: In Silico Insights towards the Identification of SARS-CoV-2 NSP13 Helicase Druggable Pockets
Source: Biomolecules. 2022 Mar 22;12(4):482. doi: 10.3390/biom12040482 (PMC9029846; doi:10.3390/biom12040482)
Supplement: Supplementary file 1 [file biomolecules-12-00482-s001.zip › biomolecules-1650620-supplementary.pdf]

In Silico Insights towards the Identification of SARS-COV2 NSP13 helicase Druggable Pockets

Federico Ricci, Rosaria Gitto, Giovanna Pitasi and Laura De Luca*

Department of Chemical, Biological, Pharmaceutical, and Environmental Sciences, University of Messina, 98166 Messina, Italy;

Corresponding: laura.deluca@unime.it; Tel.: +39-090-676-6410 (L.D.L.)

Table of contents

TableS1 Ramachandran plot for nsp13-1 and nsp13-2 extracted from PDB 6XEZ, 7CXM and 7CXN S2

TableS2 Ensemble-consensus binding-site prediction report S4

TableS3 List of the protein residues forming the identified binding sites defined to include residues within 10 Å of any centroid of Fpocket results for each protein S5

Table S1 . Ramachandran plot for nsp13-1 and nsp13-2 extracted from PDB 6XEZ, 7CXM and 7CXN

Protein	Ramachandran Plot	A	B
6XEZ, chain E (nsp13_1)	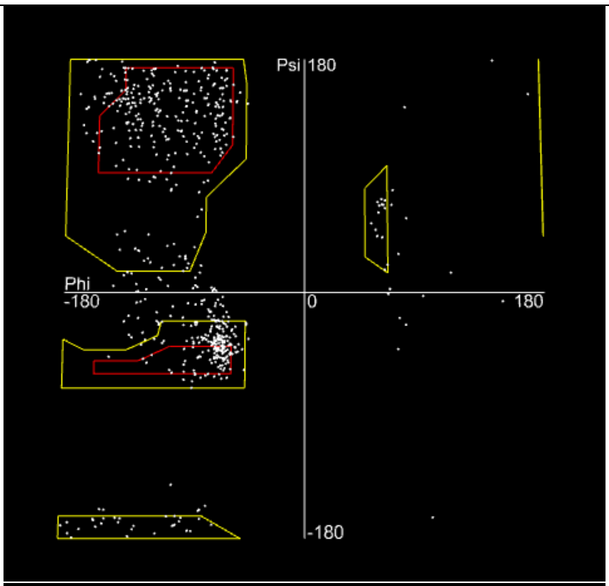	81,4 %	45,6
6XEZ, chain F (nsp13_2)	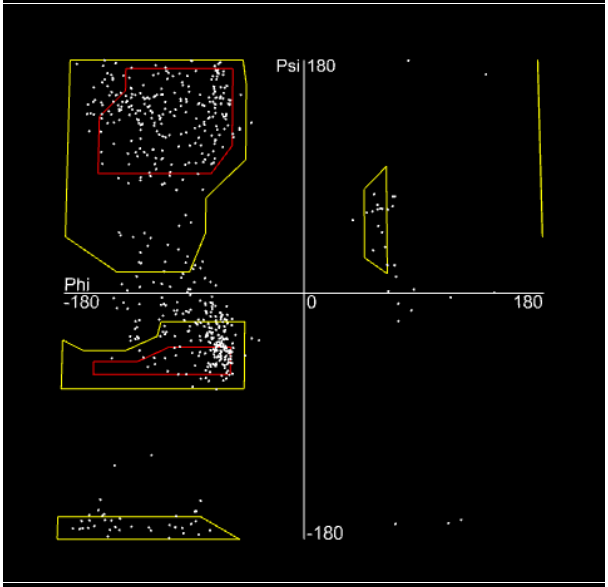	76,68 %	42,11%
7CXM, chain E (nsp13_1)	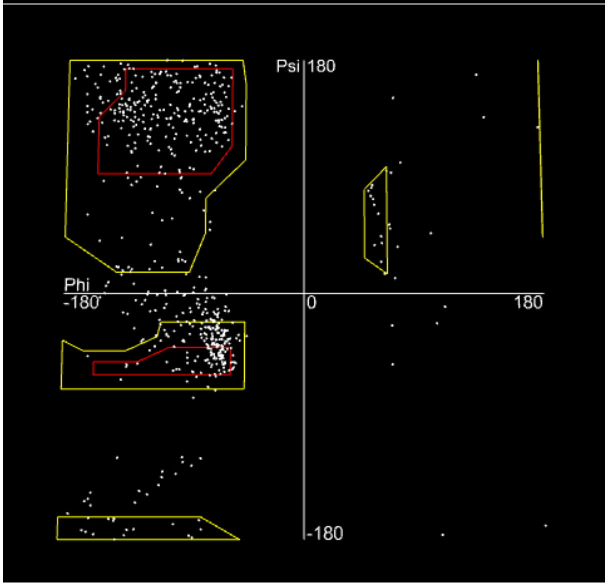	77,85%	43,79 %

7CXM, chain F (nsp13_2)	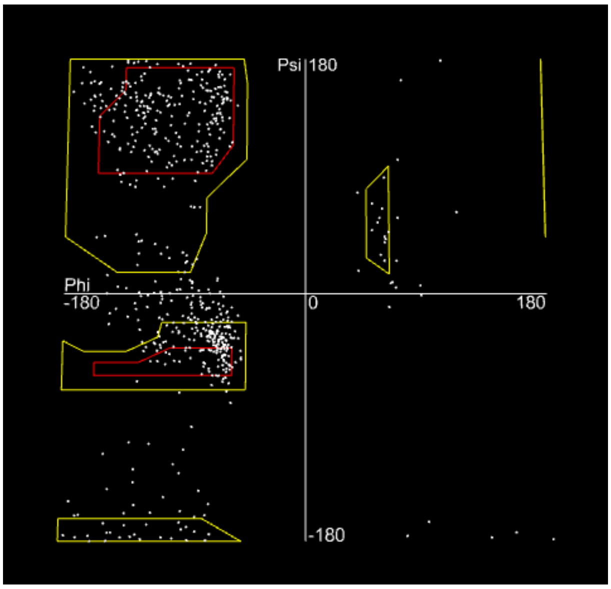	72,5%	37,58%
7CXN, chain E (nsp13_1)	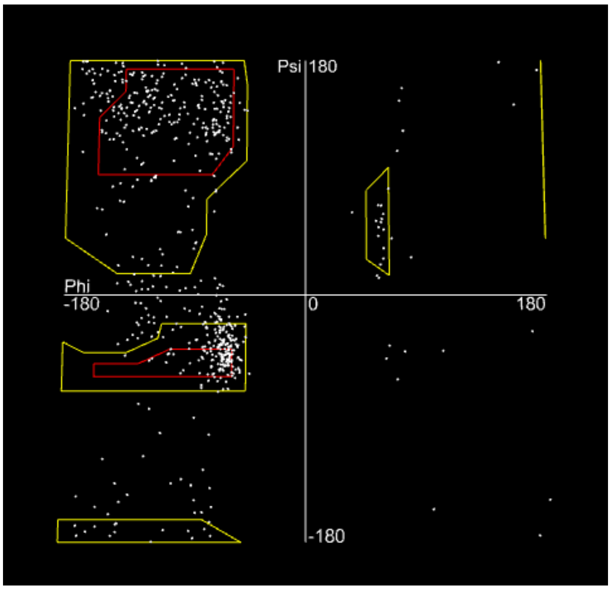	79,5%	44,3%
7CXN, chain F (nsp13_2)	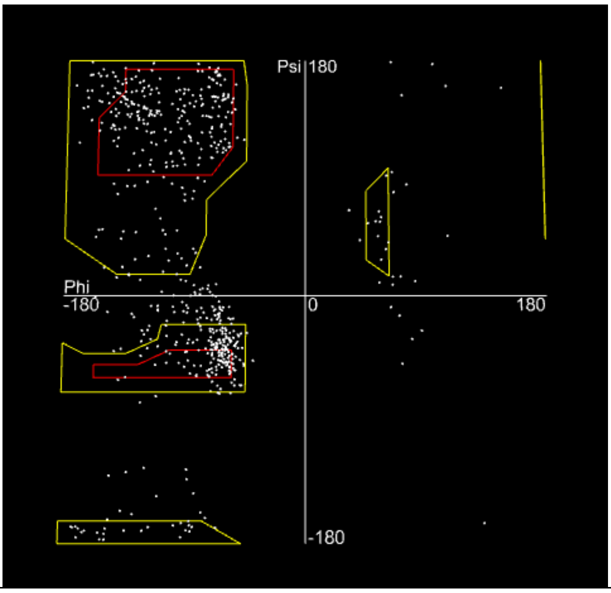	76,2%	41,6%

A: % residues belong to the most favored regions (red and yellow boundaries)
B: % correspond to conformations where there are no steric clashes (red boundary)

Table S2 . Ensemble-consensus binding-site prediction report.

	Sitemap						Fpocket						FTMap						LigandScout					
Ranking	6XEZ_E		7cxm_E		7cxn_E		6XEZ_E		7cxm_E		7cxn_E		6XEZ_E		7cxm_E		7cxn_E		6XEZ_E		7cxm_E		7cxn_E	
Position	Site	DScore	Site	DScore	Site	DScore	Site (STP)	DScore	Site (STP)	DScore	Site (STP)	DScore	Site (Cluster)	Size	Site (Cluster)	Size	Site (Cluster)	Size	Site	Y/N/ Trh val.	Site	Y/N/ Trh val.	Site	Y/N/ Trh val.
II	1	1.004	3; 1	0.993; 0.944	3; 1; 4	1.031; 0.986	28; 2; 23	0.061; 0.001; 0.002	41; 2; 43; 37	0.037; 0.003; 0.008; 0.001	18; 2; 16	0.446; 0.625; 0.070	4; 0; 9	5; 6; 16; 3	10; 4; 2; 1	3; 8; 10; 13	10; 9; 4; 3	4; 5; 8; 9	2; 8; 9	Y; 0.27; N	1; 3	Y; 0.27	1;3	0.29; 0.26
V	2	0.917	1; 4	0.944	5; 1	0,645	38; 41	0.005; 0.000	2	0.003	7	0.006	3	9					6	0,28	5	N	7	N
I	3	1.226	2	1.071	2	1,098	6	0.976	3	0.976	1	0.987	5; 12; 11	6; 2; 2	11	2	1	10	1	Y	1	Y	4; 6	0.26; N
IV	4	0.989	1	0.944	1	0,986	46	0.001; 0.002	45; 35	0.000; 0.000	43	0.001	7; 2; 1; 13	5; 12; 8; 1	0; 3	17; 10	13; 8; 6; 5; 11; 0	2; 5; 8; 8; 3						
III	5	0.946	2	1.071	2	1,098	19; 6	0.074; 0.976	3	0.976	1	0.987	11	2	11	2	1	10	4	N	1	Y	4	0.26
VI			5	0.673			10	0.003	46	0.001	17	0.003							7	N	6	N	8	N

	Sitemap						Fpocket						FTMap						LigandScout					
Ranking	6XEZ_F		7cxm_F		7cxn_F		6XEZ_F		7cxm_F		7cxn_F		6XEZ_F		7cxm_F		7cxn_F		6XEZ_F		7cxm_F		7cxn_F	
Position	Site	DScore	Site	DScore	Site	DScore	Site (STP)	DScore	Site (STP)	DScore	Site (STP)	DScore	Site (Cluster)	Size	Site (Cluster)	Size	Site (Cluster)	Size	Site	Y/N/ Trh val.	Site	Y/N/ Trh val.	Site	Y/N/ Trh val.
I	1	0.924	2; 1; 4	0.654; 0.967; 0.880	2; 1	0.794; 0.982	12; 38; 16S;	0.519; 0.001; 0.000; 0.079	1; 40; 33; 38	0.749; 0.000; 0.215; 0.000	28; 24; 38S;	0.015; 0.003; 0.131	3; 1; 5; 8	9	6; 0S	4	0; 4; 8		3; 14; 9; 2	Y; N; N; Y	1; 2;	Y; 0.27	4; 7, 8; 10	0.28; N; N; N
II	2	1.037	1	0,967	1	0.982	31; 42; 44	0.033; 0.018; 0.486	13; 21; 34;45	0.006;0.000; 0.003; 0.110	6; 46; 47; 53	0.087; 0.015; 0.000	14; 6; 4; 2; 0	1; 5; 7; 12; 18	2; 3; 7	15; 13; 3	1; 11	10; 2	1;7	Y; 0.27	4.5	N, N	1	Y
III	3	1.051			4	1.082			6; 19	0.031; 0.001	11; 37	0.662; 0.025	11; 10	2; 2			6; 9	5; 3	5	Y			2	Y
VII	4	1.018			.		30	0,12	27	0	13	0							6	Y				
VIII	5	0.7			.		32	0.046	5	0.002	.								8	N	7	N	5	0.25
V			5	0.743	3	1.071	40	0.002	18; 30	0.023; 0.001	42	0,074					12	3	10	N	3	N	6	N
VI			3	0.831	1	0.982			41	0,04	54	0,023							2	Y	11	N		
IV			1	0.967	5	0.972	7	0.001	8	0,008	9	0,047			1	15	2	9	9	N	10	N	3	0.28

Combination matrix of the sites identified for the nsp13 _1 and nsp13_2 for the three pdbs (6XEZ; 7CXM and 7CXN) analyzed using Sitemap, Fpocket, FTMap and LigandScout (blue, magenta, yellow and green bordered columns respectively). Each coloured block is then shared into three other blocks, each one corresponding to one PDB and containing the sites identified by the software. For Sitemap and Fpocket was taken into account the Druggability score(Dscore) of each site, for FTMap was considered the number of probes that binded a specific site (probe cluster) and for LigandScout we measured the goodness of the sites encountered basing on whether they were recognized as druggable or not. Each consensus pocket is ranked considering all the results reported in the table and comparing them. The one with the highest degree of overlap in the data obtained is ranked as 1st while the one with the worst match in the data considered is ranked as 5th.

Table S3. List of the protein residues forming the identified binding sites defined to include residues within 10 Å of any centroid of Fpocket results for each protein.

Proteins	Ensemble Consensus sites	Residues
Nsp13-1	**I**	Cys471, Lys473, Gln492, Ile493, Val495, Val496, Arg497, Glu498, Phe499, Leu500, Thr501, Arg502, Asn503, Pro504, Trp506, Arg507, Ala509, Val510, Phe511, Ile512, Gln518, Ala522, Leu526, Gly527, Leu528, Tyr543, Val544, Ile545, Phe546, Thr547, Ile572, Leu573, Cys574, Ile575, Ile592, Arg595
	**II**	Ile109, Trp114, Asp119, Tyr120, Ile121, Ala123, Asn124, Lys131, Leu132, Phe133, Ala134, Ala135, Glu136, Thr137, Leu138, Lys139, Ala140, Thr141, Glu142, Glu143, Val232, Leu235, Thr239, Met378, Ala379, Thr380, Asn381, Tyr382, Asp383, Leu384, Ser385, Pro406, Ala407, Pro408, Arg409, Thr410, Leu411, Leu412, Leu417, Tyr421, Phe422, Ser424
	**III**	Ala 469, Cys471, Lys473, Gln492, Ile493, Gly494, Val495, Val496, Arg497, Glu498, Phe499, Leu500, Thr501, Arg502, Asn503, Pro504, Trp506, Arg507, Ala509, Val510, Phe511, Ile512, Gly527, Leu528, Tyr543, Val544, Ile545, Phe546, Thr547, Ile572, Leu573, Cys574, Ile575, Leu 590, Ile592, Arg594, Arg595
Nsp13-2	**I**	Leu 280, Gly 282, Pro 283, Pro 284, Gly 285, Thr 286, Gly 287, Lys 288, Ser 289, Hys 290, Phe 291, Ala 292, Ile 293, Ala 308, Ala 312, Ala 313, Val 314, Asp 315, Ala 316, Leu 317, Cys 318, Glu 319, Lys 320, Ala 321, Lys 323, Tyr 324, Phe 357, Val 372, Asp 374, Glu 375, Ile 376, Ile 399, Gly 400, Asp 401, Gln 404, Leu 438, Arg 443, Ser 536, Gln 537, Gly 538, Ser 539, Glu 540, Arg 567
	**II**	Trp114, Tyr120, Ala134, Ala135, Glu136, Thr137, Leu138, Lys 139, Ala140, Thr141, Glu143, Thr144, Phe145, Lys146, Asn179, Tyr180, Leu227, Ser229, Val232, Cys309, Asn361, Met378, Ala379, Thr380, Asn381, Tyr382, Asp383, Val386, Ala407, Pro408, Arg409, Thr410, Leu411, Asn423
	**III**	Lys473, Asn489, Arg490, Gln492, Ile493, Gly494, Val495, Val496, Arg497, Glu498, Phe499, Leu500, Thr501, Arg502, Asn503, Trp506, Arg507, Ala509, Val510, Phe511, Ile512, Ser513, Gln518, Asn519, Val521, Ala522, Ser523, Ile525, Leu526, Gly527, Leu528, Pro529, Thr530, Ile545, Phe546, Thr547, Leu573, Ile575, Arg595
	**IV**	Pro284, Gln404, Leu405, Ile448, Lys508, Val510, Phe511, Ile512, Ser513, Asn519, Pro529, Thr530, Gln531, Thr532, Val533, Asp534, Ser535, Ser536, Gln537, Gly538, Ser539, Glu540, Tyr541, Asp542, Tyr543, Val544, Ile545, Phe546, Arg560, Phe561, Asn562, Val563, Ala564, Ile565, Thr566, Arg567, Ala568, Lys569, Gly571, Ile572
